# Comparison of 24-month treatment outcomes between as-needed treatment and switching to treat-and-extend in type 3 macular neovascularization

**DOI:** 10.1038/s41598-022-25860-5

**Published:** 2022-12-29

**Authors:** Jae Hui Kim, Jong Woo Kim, Chul Gu Kim

**Affiliations:** Department of Ophthalmology, Kim’s Eye Hospital, #156 Youngdeungpo-dong 4Ga, Youngdeungpo-gu, 150-034 Seoul, South Korea

**Keywords:** Macular degeneration, Retinal diseases

## Abstract

This study aimed to compare 24-month treatment outcomes between patients with type 3 macular neovascularization (MNV) treated using an as-needed regimen and those who switched to treat-and-extend (TAE). This retrospective study included 32 patients who were initially treated with an as-needed regimen but switched to TAE (TAE group) and 74 patients who were treated with an as-needed regimen throughout the follow-up period (as-needed group). The number of anti-vascular endothelial growth factor (VEGF) injections and degree of change in best-corrected visual acuity (BCVA) over 24 months were compared between the two groups. The incidence of fibrotic scarring, tears of the retinal pigment epithelium (RPE), and subretinal hemorrhage was also evaluated. The number of anti-VEGF injections was higher in the TAE group (mean: 11.7) than in the as-needed group (mean: 6.9; *P* < 0.001). The BCVA outcome (measured using the mean logarithm of the minimal angle of resolution [logMAR]) was significantly better in the TAE group (mean improvement of logMAR 0.15) than in the as-needed group (mean deterioration of logMAR 0.15). The incidence of fibrotic scarring (6.3% vs. 18.9%), RPE tears (3.1% vs. 6.8%), and subretinal hemorrhage (0% vs. 9.5%) was relatively lower in the TAE group. Treatment outcomes of the TAE group were better than those of the as-needed group, suggesting that switching to the TAE regimen would be a useful approach for patients with type 3 MNV requiring efficient treatment.

## Introduction

Type 3 macular neovascularization (MNV)^1^, also called retinal angiomatous proliferation^2^, is a subtype of neovascular age-related macular degeneration (AMD) characterized by intraretinal neovascularization. Patients with type 3 MNV have characteristics distinct from those of patients with other subtypes of neovascular AMD, including old age^3^, thin choroid^4^, high incidence of reticular pseudodrusen^5^, and a high risk of fellow-eye involvement^6^. In particular, the risk of retinal pigment epithelial (RPE) atrophy is relatively higher in type 3 MNV^7,8^.

Intravitreal anti-vascular endothelial growth factor (VEGF) offers an effective treatment for type 3 MNV^9–14^. However, the treatment outcome is poor in advanced disease^10^. In addition, some patients can experience abrupt visual loss within a short period of time^15,16^. Due to this relentless nature of the disease, Engelbert et al. suggest that the as-needed regimen, which is characterized by “wait and watch, treat if necessary” might not be ideal for type 3 MNV^9^. As a result, an early study to evaluate the efficacy of the treat-and-extend (TAE) regimen was performed for type 3 MNV^9^. The TAE regimen is a widely-used treatment regimen for neovascular AMD that is characterized by continuous anti-VEGF injections with adjustment of the injection interval^17^. The outcome of the TAE regimen is reported to be generally superior to that of the as-needed regimen^18^. In addition, switching to the TAE regimen in patients initially treated with the as-needed regimen was found to be effective in stabilizing visual acuity (VA) and macular thickness^19,20^.

Previous studies have demonstrated that the TAE regimen is effective for type 3 MNV^11,14^. However, the TAE regimen usually has a higher injection frequency than the as-needed regimen. Several investigators have suggested that the development of RPE atrophy is influenced by injection frequency^21^. For this reason, there has been some debate as to whether the as-needed or TAE regimen is safer and more effective for patients with type 3 MNV^17^. To date, many studies have reported on treatment outcomes of type 3 MNV using the as-needed or TAE regimen. However, limited knowledge is available regarding a direct comparison between the two. Previously, we attempted to address this issue^20^. However, meaningful conclusions could not be drawn because of the small sample size.

In the present study, 24-month treatment outcomes were compared between patients with type 3 MNV treated using the as-needed regimen and those initially treated using the as-needed regimen but switched to the TAE regimen during treatment. Outcomes were also compared with those of patients who did not experience lesion reactivation after the initial treatment.

## Materials and methods

This retrospective observational study was conducted at a single center (Kim’s Eye Hospital, Seoul, South Korea). The study was approved by the Institutional Review Board (IRB) of Kim’s Eye Hospital and conducted in accordance with the tenets of the Declaration of Helsinki. Due to the retrospective nature of this study, the need for informed consent was waived (Kim’s Eye Hospital IRB, Seoul, South Korea).

### Patients

This study included treatment-naïve patients who were diagnosed with type 3 MNV between January 2015 and December 2019 and who were initially treated with three loading injections using ranibizumab (0.5 mg/0.05 mL of Lucentis®; Genentech Inc., San Francisco, CA, USA) or aflibercept (2.0 mg/0.05 mL of Eylea®; Regeneron, Tarrytown, NY, USA). Exclusion criteria were as follows: (1) less than 24 months of follow-up, (2) presence of definite chorioretinal anastomosis on fundus photography at diagnosis, (3) previous history of vitreoretinal or glaucoma surgery, and (4) discontinued treatment immediately after three loading injections due to poor treatment outcomes. Some patients included in previous studies by our group^8,20^ were also included in the present study. When both eyes met the inclusion criteria, eyes with prior symptoms were included in the study.

At the initial diagnosis, all patients underwent fluorescein angiography, indocyanine green angiography, and optical coherence tomography (OCT) examinations. Patients were diagnosed with type 3 MNV when characteristic intraretinal hyperreflective lesions with surrounding retinal edema were noted on OCT, accompanied by hyperfluorescence on angiography. The staging of type 3 MNV was performed based on the suggestion by Su et al.^22^: stage 1 = intraretinal hyperreflective lesion with surrounding edema without involving external limiting membrane or ellipsoid zone; stage 2 = intraretinal hyperreflective lesion with surrounding edema with external limiting membrane or ellipsoid zone involvement; stage 3 = presence of subretinal fluid or pigment epithelial detachment.

### Treatment

All patients received three anti-VEGF injections at monthly intervals as an initial treatment. Subsequently, as-needed regimen-based retreatment was performed. During the first 12 months, patients were followed-up at 1- or 2-month intervals. Subsequently, the follow-up interval was extended to 3–4 months when there was no lesion reactivation. In selected cases, the treatment regimen was changed to TAE when the treating physician determined that a more effective treatment was required to preserve vision.

There are no strict guidelines for switching treatment regimens. In general, switching was conducted for the following reasons: (1) when definite visual deterioration was noted with lesion reactivation, (2) poor VA in the fellow-eye, (3) when the fellow-eye was also diagnosed with neovascular AMD, (4) prechoroidal cleft development, and (5) when the patient requested more effective treatment. The TAE protocol was slightly modified based on the originally suggested protocol^9^. The injection interval was adjusted to between 2 and 3 weeks, with the longest interval of 3 to 4 months. In retreatment, bevacizumab (1.25 mg/0.05 mL of Avastin®; Genentech Inc., San Francisco, CA, USA) was also used along with ranibizumab or aflibercept.

Treatment was discontinued when the treating physician determined that the additional treatment was not beneficial. After treatment discontinuation, patients were followed-up with at intervals of 2 to 6 months without additional injections.

### Outcome measures

Participants were classified into three groups according to the following criteria: TAE group = patients who were initially treated using the as-needed regimen but switched to the TAE regimen during the follow-up period; as-needed group = patients who were treated using the as-needed regimen during the 24-month follow-up period and who experienced at least one lesion reactivation after initial loading injections; no-reactivation group = patients who did not experience lesion reactivation after initial loading injections.

The following baseline parameters were compared among the three groups: age, sex, stage of type 3 MNV, central retinal thickness (CRT), subfoveal choroidal thickness (< 150 µm vs. ≥ 150 µm), and anti-VEGF drug used for the initial treatment (ranibizumab vs. aflibercept). To compare treatment outcomes, the following parameters were compared among the three groups: number of anti-VEGF injections, degree of change in best-corrected visual acuity (BCVA) and CRT over 24 months, incidence of fovea-involving RPE atrophy or fibrotic scarring, subretinal hemorrhage greater than three-disc areas, and RPE tearing. When the patient failed to visit the hospital at 24 months, data from the closest hospital visit were used for the analysis. CRT was defined as the average retinal thickness of the central 1 mm region as defined by the Early Treatment Diabetic Retinopathy Study (ETDRS).

To evaluate the difference in visual outcomes between patients initially treated with ranibizumab (ranibizumab group) and those with aflibercept (aflibercept group), the degree of change in BCVA was compared between the two groups. This additional analysis was performed within the as-needed, TAE, and no-reactivation groups. The number of fluid recurrences requiring anti-VEGF injection or shortening of the injection interval was additionally counted in the TAE and as-needed groups. Persistent fluid and/or hemorrhages after treatment discontinuation were not counted in this analysis.

VAs measured when the treatment was changed from the as-needed regimen to the TAE regimen in the TAE group were compared with those measured at the corresponding time point in the as-needed group (at the 11 month follow-up visit or closest follow-up to 11 months). Since measurement of BCVA was not routinely performed during every follow-up visit, BCVAs as well as corrected VAs (VA was measured with each patient wearing eyeglasses) were used for this analysis.

### Statistical analysis

Data are presented as mean ± standard deviation or number (%), where applicable. VA was measured using a decimal VA chart and transformed to logarithm of the minimal angle of resolution (logMAR) values for analysis. The counting finger and hand motion VAs were converted to logMAR values of 2 and 3, respectively. Comparisons of BCVA and CRT among the three groups were performed using the Kruskal–Wallis test, and individual comparisons were performed using the Mann–Whitney *U* test with Bonferroni’s correction. Comparisons of other parameters among the three groups were performed using the Kruskal–Wallis, chi-square, or Fisher’s exact tests. Comparisons between two groups were performed using the Mann–Whitney *U* test. Statistical analyses were performed using a commercially available software package (Statistical Package for the Social Sciences for Windows, version 21.0; IBM, Armonk, NY, USA). Statistical significance was set at *P* < 0.05.

## Results

In total, 132 patients were included in this study (Table 1).

**Table 1:** Baseline characteristics of patients included in the study (n = 132).

Characteristic	
Age	75.8 ± 5.8
**Sex**	
Men	19 (14.4%)
Women	113 (85.6%)
Stage of disease*	
Stage 2	34 (25.8%)
Stage 3	98 (74.2%)
Best-corrected visual acuity, logMAR	0.60 ± 0.42
Central retinal thickness, µm	438.8 ± 131.9
**Subfoveal choroidal thickness**	
< 150 µm	73 (55.3%)
≥ 150 µm	59 (44.7%)
**Type of anti-VEGF agent used for initial loading injections**	
Ranibizumab	81 (61.4%)
Aflibercept	51 (38.6%)

Data are presented as mean ± standard deviation or number (%), where applicable.

*logMAR* logarithm of minimal angle of resolution; *VEGF* vascular endothelial growth factor.

*Disease stage was classified based on the method proposed by Su et al.

There were 19 (14.4%) men and 113 (85.6%) women in the study sample. The mean age of the patients was 75.8 ± 5.7 years (range, 60–92). The TAE group had 32 patients (24.2%), as-needed had 74 (56.1%), and the remaining 26 (19.7%) patients were included in the no-reactivation group. The total study period was 24 months in all three groups. In the TAE group, the as-needed phase was the first 10.8 ± 5.2 months after diagnosis and the TAE phase was the next 13.2 ± 5.2 months.

For the initial treatment, 81 patients were treated using ranibizumab and 51 were treated using aflibercept. Among them, 74 were treated using ranibizumab throughout the entire follow-up period and 40 using aflibercept throughout this entire period. Mixed use of anti-VEGF agents took place for the remaining 18 patients. Among these 18 patients, 13 received at least one bevacizumab injection.

The results of comparisons of baseline characteristics among the three groups are presented in Table 2.

**Table 2:** Comparison of baseline characteristics among the TAE, as-needed, and no-reactivation groups.

Characteristic	TAE group (n = 32)	As-needed group (n = 74)	No-reactivation group (n = 26)	*P*-value
Age	75.0 ± 6.7	76.0 ± 5.9	76.2 ± 3.9	0.647^†^
Sex				0.226^‡^
Men	5 (15.6%)	13 (17.6%)	1 (3.8%)	
Women	27 (84.4%)	61 (82.4%)	25 (96.2%)	
Stage of disease*				0.241**
Stage 2	5 (15.6%)	20 (27.0%)	9 (34.6%)	
Stage 3	27 (84.4%)	54 (73.0%)	17 (65.4%)	
Best-corrected visual acuity, logMAR (Snellen equivalents)	0.60 ± 0.39 (20/79)	0.56 ± 0.39 (20/72)	0.71 ± 0.53 (20/102)	0.614^†^
Central retinal thickness, µm	462.3 ± 161.3	440.9 ± 122.1	403.8 ± 115.6	0.340^†^
Subfoveal choroidal thickness				0.216**
< 150 µm	20 (62.5%)	36 (48.6%)	17 (65.4%)	
≥ 150 µm	12 (37.5%)	38 (51.4%)	9 (34.6%)	
Type of anti-VEGF agent used for initial loading injections				0.002**
Ranibizumab	28 (87.5%)	40 (54.1%)	13 (50.0%)	
Aflibercept	4 (12.5%)	34 (45.9%)	13 (50.0%)	
Type of anti-VEGF agent used throughout the entire treatment period				0.001^‡^
Ranibizumab only	25 (78.1%)	36 (48.7%)	13 (50.0%)	
Aflibercept only	3 (9.4%)	24 (32.4%)	13 (50.0%)	
Ranibizumab and aflibercept	3 (9.4%)	2 (2.7%)	0	
Addition of bevacizumab	1 (3.1%)	12 (16.2%)	0	

Data are presented as mean ± standard deviation or number (%), where applicable.

*logMAR* logarithm of minimal angle of resolution; *TAE* treat-and-extend; *VEGF* vascular endothelial growth factor.

^†^ Statistical analysis with the Kruskal–Wallis test.

^‡^ Statistical analysis with the Fisher’s exact test.

* Disease stage was classified based on the method proposed by Su et al.

** Statistical analysis with the chi-square test.

**Figure 1:**
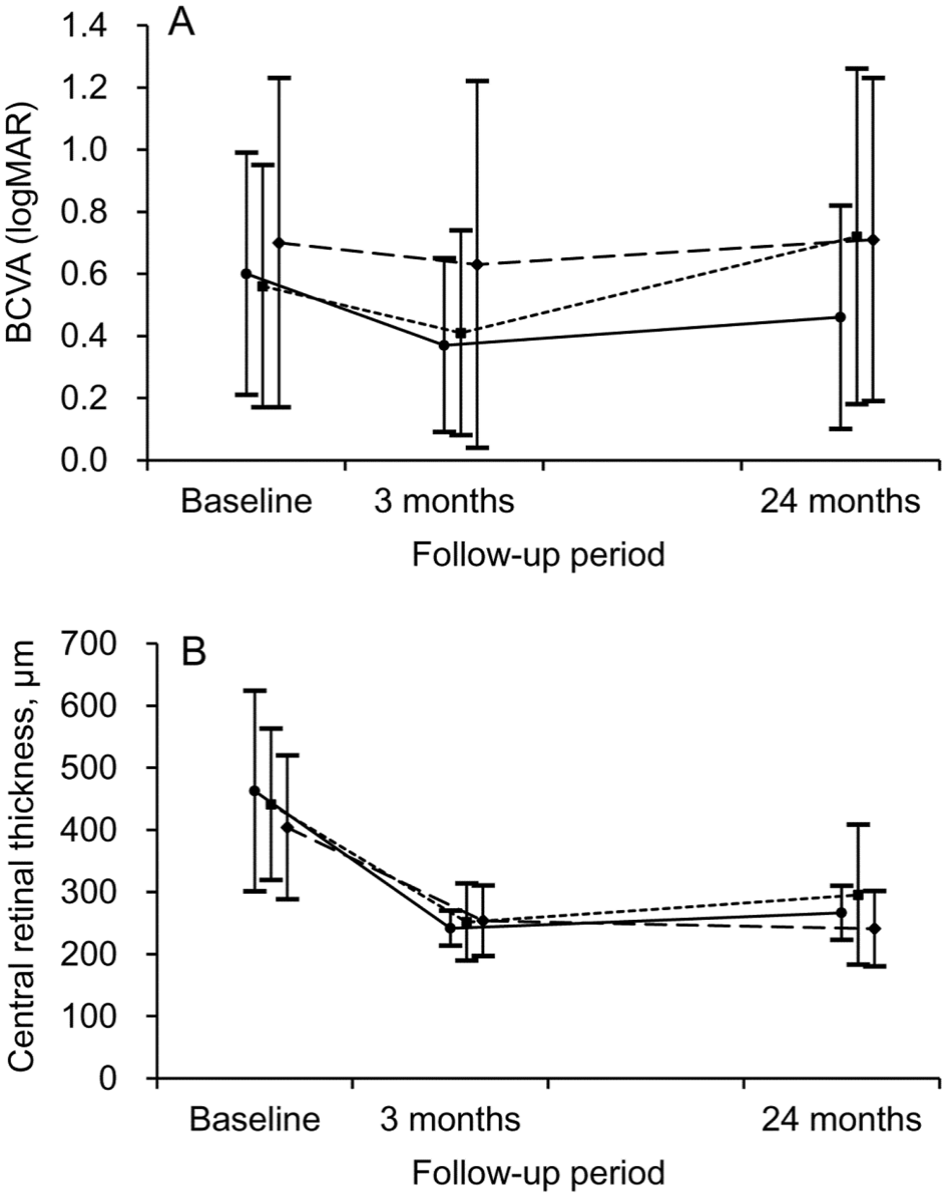
Time-dependent changes in best-corrected visual acuity (BCVA) (**A**) and central retinal thickness (**B**), according to the treatment groups: treat-and-extend group = closed circle, solid line (n = 32); as-needed group = closed square, dotted line (n = 74); no-reactivation group = closed diamond, dashed line (n = 26). Note that BCVA markedly deteriorates after 3 months in the as-needed group, whereas the degree of deterioration is relatively lower in the other two groups (**A**). *logMAR* logarithm of minimal angle of resolution.

The mean number of anti-VEGF injections was 11.7 ± 2.8 in the TAE group, 6.9 ± 2.1 in the as-needed group, and 3.0 ± 0 in the no-reactivation group. The number was significantly higher in the TAE group than in the as-needed (*P* < 0.001) or no-reactivation (*P* < 0.001) groups. The number of fluid recurrences after the initial three loading injections was 4.0 ± 2.1 in the as-needed group and 4.4 ± 2.4 in the TAE group during the 24 month follow-up period. In the TAE group, the mean logMAR BCVA values were 0.60 ± 0.39 (Snellen equivalent = 20/79) at baseline, 0.37 ± 0.28 (20/46) at 3 months, and 0.46 ± 0.36 (20/57) at 24 months (Fig. 1A).

In the as-needed group, the mean logMAR BCVA values were 0.56 ± 0.39 (20/72) at baseline, 0.41 ± 0.33 (20/57) at 3 months, and 0.72 ± 0.54 (20/104) at 24 months. In the no-reactivation group, the values were 0.70 ± 0.53 (20/100) at baseline, 0.63 ± 0.59 (20/85) at 3 months, and 0.71 ± 0.52 (20/102) at 24 months.

After 24 months, a mean improvement of logMAR 0.15 ± 0.34 in BCVA was noted in the TAE group, a mean deterioration of logMAR 0.15 ± 0.53 in BCVA was noted in the as-needed group, and a mean deterioration of logMAR 0.01 ± 0.29 in BCVA was noted in the no-reactivation group. There was a significant difference in the degree of change among the three groups (*P* = 0.013). The difference between the TAE and as-needed groups was statistically significant (*P* = 0.012). However, there was no significant difference in degree of change between the TAE and no-reactivation groups (*P* = 0.147) and between the as-needed and no-reactivation groups (*P* = 1.000). During the 24-month follow-up period, five-line deterioration or greater in BCVA was noted in 1 patient (3.1%) in the TAE group, 12 (16.2%) in the as-needed group, and 2 (7.7%) in the no-reactivation group.

In the TAE group, the mean CRT values were 462.3 ± 161.3 µm at baseline, 241.5 ± 28.5 µm at 3 months, and 266.3 ± 43.5 µm at 24 months (Fig. 1B). In the as-needed group, the values were 440.9 ± 122.1 µm at baseline, 251.3 ± 62.0 µm at 3 months, and 295.3 ± 112.8 µm at 24 months. In the no-reactivation group, the values were 403.8 ± 115.6 µm at baseline, 253.4 ± 56.9 µm at 3 months, and 240.7 ± 60.7 µm at 24 months. After 24 months, the degree of mean CRT reduction was 195.9 ± 149.2 in the TAE group, 145.6 ± 158.1 µm in the as-needed group, and 163.1 ± 106.0 µm in the no-reactivation group. There was no significant difference in the degree of CRT change among the three groups (*P* = 0.488).

The incidences of fovea-involving RPE atrophy, fibrotic scarring, subretinal hemorrhage, and RPE tears in the TAE, as-needed, and no-reactivation groups are presented in Table 3.

**Table 3:** Comparison of incidences of pathologic fundus findings and hemorrhage development at 24 month follow-up among the TAE, as-needed, and no-reactivation groups.

Characteristic	TAE group (n = 32)	As-needed group (n = 74)	No-reactivation group (n = 26)	*P*-value
Fovea-involving RPE atrophy	6 (18.8%)	14 (18.9%)	16 (61.5%)	< 0.001*
Fovea-involving fibrotic scar	2 (6.3%)	14 (18.9%)	2 (7.7%)	0.161^†^
RPE tear	1 (3.1%)	5 (6.8%)	0	0.536^†^
Subretinal hemorrhage^‡^	0	7 (9.5%)	0	0.069^†^

Data are presented as number (%).

*TAE* treat-and-extend; *RPE* retinal pigment epithelial.

* Statistical analysis with the chi-square test.

^†^ Statistical analysis with the Fisher’s exact test.

^‡^ Incidence of extent of hemorrhage with an area greater than three discs was evaluated.

**Figure 2:**
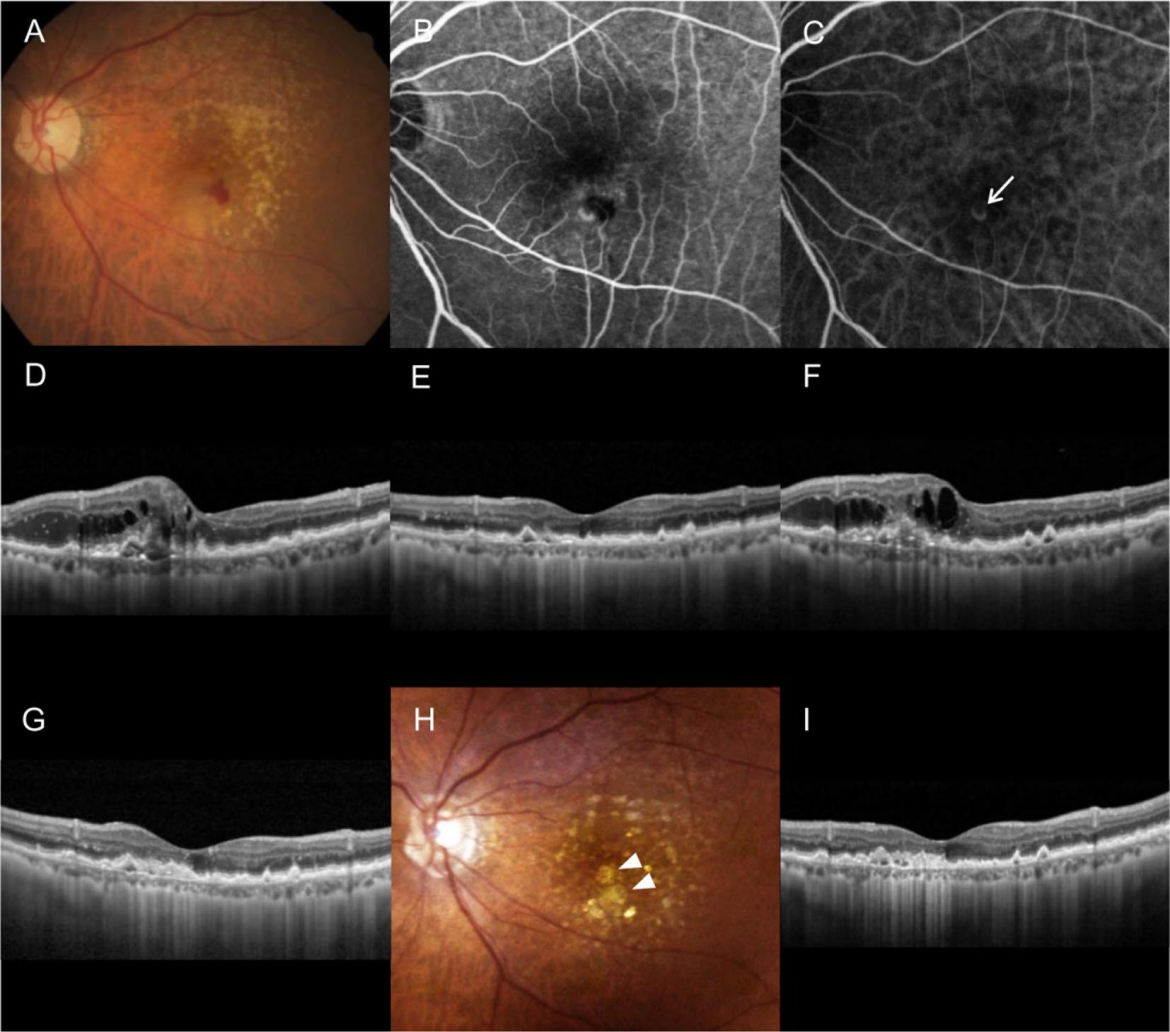
A representative case showing the clinical course of a patient in the treat-and-extend (TAE) group. An 80-year-old patient diagnosed with type 3 macular neovascularization (MNV) (**A**–**D**). The best-corrected visual acuity (BCVA) is 20/100. After three monthly ranibizumab injections, retinal edema completely resolved (**E**). Seven months after the last injection, lesion reactivation is noted (**F**) and the treatment method was switched to the TAE regimen. The retinal edema is once again completely resolved after the fourth injection (**G**). During the 24 month follow-up period, 14 ranibizumab injections were performed. At 24 months (**H**, **I**), there is no lesion reactivation and the BCVA is 20/50. On fundus photography, retinal pigment epithelial atrophy is noted ((**H)**, arrowheads). (**A**, **H)** = fundus photography; (**B)** = fluorescein angiography; (**C)** = indocyanine green angiography; (**D**-**G)**, (**I)** = optical coherence tomography. The arrow in panel (**C)** indicates a type 3 MNV lesion.

**Figure 3:**
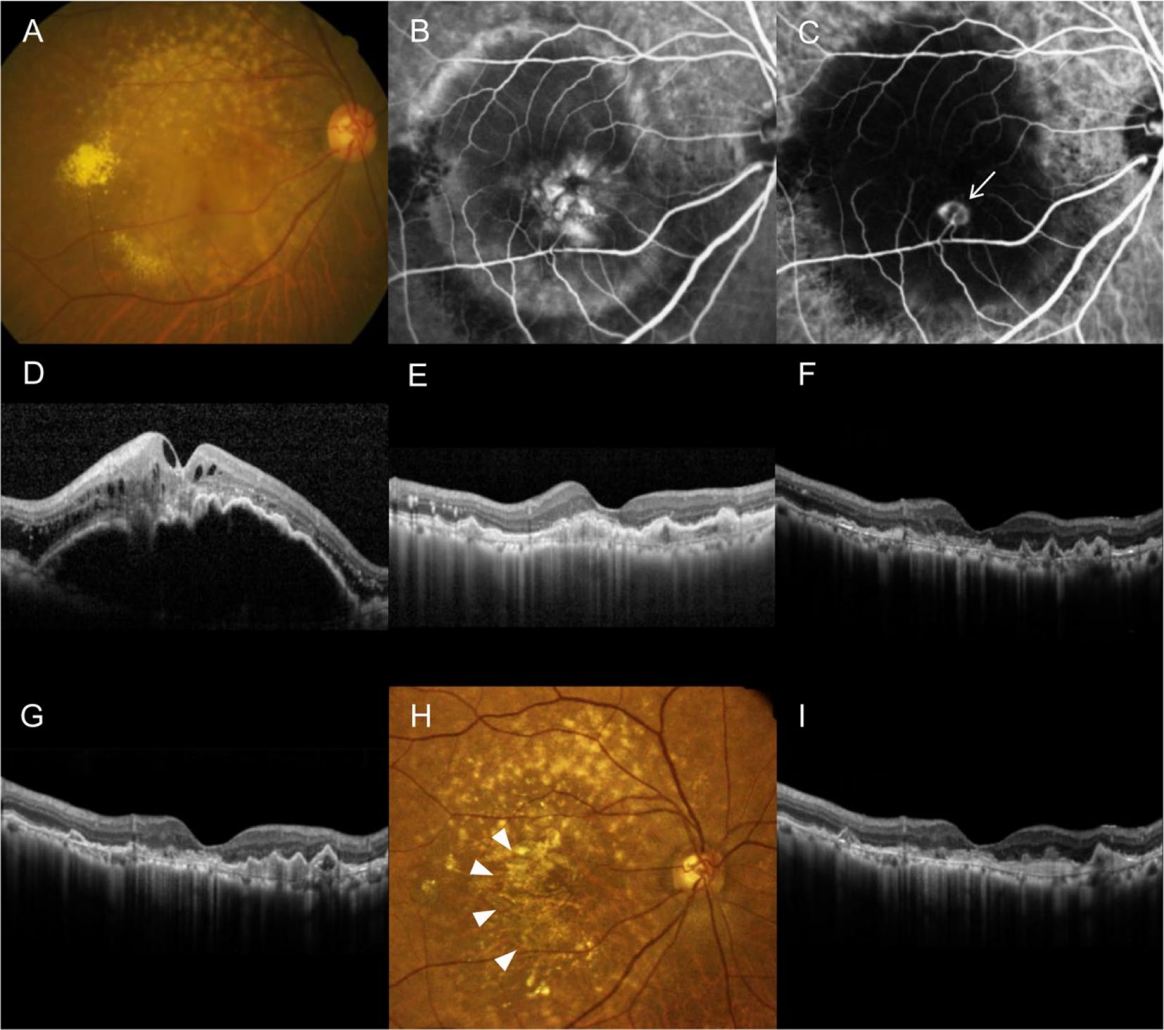
A representative case showing the clinical course of a patient in the no-reactivation group. A 76-year-old patient diagnosed with type 3 macular neovascularization (MNV) (**A**–**D**). The best-corrected visual acuity (BCVA) is 20/400. After three monthly aflibercept injections, retinal edema completely resolved (**E**). Optical coherence tomography (OCT) images taken at 11 months (**F**) and 18 months (**G**) show no evidence of lesion reactivation. No additional injection was required during the 24-month follow-up period. At 24 months (**H**, **I**), there is no lesion reactivation and the BCVA is 20/400. On fundus photography, retinal pigment epithelial atrophy is noted ((**H)**, arrowheads). (**A)**, (**H)** = fundus photography; (**B**) = fluorescein angiography; (**C)** = indocyanine green angiography; (**D**-**G)**, (**I)** = OCT. The arrow in panel (**C)** indicates a type 3 MNV lesion.

There was a significant difference in the incidence of fovea-involving RPE atrophy among the TAE (6 patients, 18.8%), as-needed (14 patients, 18.9%), and no-reactivation (16 patients, 61.5%) groups (*P* < 0.001). The incidences of fibrotic scarring, subretinal hemorrhage, and RPE tears were relatively higher in the as-needed group than in the other groups. However, this difference was not significant. Figures 2 and 3 show representative cases of patients in the TAE and no-reactivation groups, respectively.

There were no significant differences in the degree of change in BCVA between the ranibizumab (n = 28) and aflibercept (n = 4) groups in the TAE group (*P* = 0.978), in the as-needed group (ranibizumab, n = 40; aflibercept, n = 34; *P* = 0.943), or in the no-reactivation group (ranibizumab, n = 13; aflibercept, n = 13; *P* = 0.059).

In the TAE group, the mean logMAR corrected VA value was 0.44 ± 0.22 when the treatment method was changed from the as-needed regimen to the TAE regimen (mean 10.8 months). In the as-needed group, the value was 0.52 ± 0.35 at the mean of 11.4 ± 0.8 months (*P* = 0.184).

## Discussion

In previous studies, both the as-needed regimen^10,13^ and TAE regimen^9,11,14^ were effective in treating type 3 MNV. In a prospective study by Shin et al., using the as-needed regimen, a mean of 7.7 ranibizumab injections were performed during a 24 month treatment period^10^. VA significantly improved from a mean of 48.7 ETDRS letters to a mean of 56.3 letters. In a study by Kim et al. with a 12 month follow-up, VA significantly improved in patients with stage 2 disease (mean logMAR 0.61 to 0.46), but not in those with stage 3 disease (mean logMAR 0.67 to 0.70)^13^. In addition, stage progression was noted in 14.3% of patients, suggesting that long-term treatment outcomes can be influenced by this stage progression^13^.

In general, visual outcomes of patients treated with the TAE regimen were relatively better than visual outcomes of those treated with the as-needed regimen. In an early TAE study by Engelbert et al., the mean baseline VA was 20/80, improved to 20/40 at 1 month, and was maintained throughout the 36 month follow-up period^9^. The mean retinal thickness decreased from 320 µm to 180–230 µm, and was maintained during the study period. Based on this observation, the authors concluded that the TAE regimen delivers promising outcomes in type 3 MNV^6^. In a study by Matsumoto et al. VA significantly improved from mean logMAR 0.57 at baseline to mean logMAR 0.32 at 12 months, with a significant decrease in retinal thickness^11^. In a more recent prospective clinical trial by Arias et al. a mean improvement of 10.5 ETDRS letters in BCVA was noted at week 52 with a significant decrease in retinal thickness. Furthermore, the proportion of patients with intraretinal/subretinal fluid decreased from 87.5% at baseline to 11.5% at week 52^14^.

Although the TAE regimen is an effective and efficient method, it may result in unnecessary injections in some patients. It is well-known that there is no lesion reactivation after initial treatment in some neovascular AMD patients^23,24^. In type 3 MNV, this proportion is reported to be 19% during a mean follow-up of 27.5 months^25^. In fact, 19.7% of patients in the present study did not experience lesion reactivation during the 24 month follow-up period. For these patients, the as-needed basis of retreatment is more appropriate than the TAE regimen. However, it is difficult to predict lesion reactivation accurately. In addition, type 3 MNV may undergo a relentless course^9^. Thus, using an as-needed regimen for a long period may not be a plausible approach to preserve vision in type 3 MNV. In a study by Hatz et al. investigating patients with neovascular AMD, switching from an as-needed regimen to a TAE regimen improved and stabilized both anatomical and functional outcomes^19^. In addition, there were no vision-threatening events during the TAE phase^19^. We observed a similar outcome in our previous study on type 3 MNV^20^.

In the present study, the 24 month visual outcome of patients switching to the TAE regimen during treatment was relatively favorable with a mean visual improvement of 1.5 lines, despite the long-term follow-up period. In addition, development of fibrotic scars, RPE tears, or subretinal hemorrhage was noted in only a small proportion of patients. This treatment outcome was better than that of patients treated with the as-needed regimen throughout the follow-up period. Moreover, it was relatively better in patients who did not experience lesion reactivation after the initial treatment. Based on these results, we suggest that using an as-needed regimen during the early treatment period followed by switching to the TAE regimen would be a useful treatment strategy to achieve efficiency without sacrificing efficacy. In the present study, the actual mean TAE phase was 13.2 months in the TAE group. Thus, this should be considered when interpreting the study results.

When compared with previous studies that opted for the TAE regimen right after diagnosis, the visual outcome in the TAE group was similar to that of a study by Mrejen et al., in which approximately one line of visual improvement was noted at 24 months^26^. Additionally, it was relatively inferior to that of a study by Engelbert et al., in which VA improved from 20/80 at baseline to approximately 20/40 at 2 years^9^.

The TAE regimen is an effective treatment method with a promising visual outcome. However, in a clinical setting, applying the TAE regimen right after the loading injections is sometimes not possible due to various reasons, such as affordability, fear of intraocular injections, and public or private health insurance policies. In these situations, starting with an as-needed regimen followed by switching to a TAE regimen can be a useful alternative approach. We postulate that patients with a low risk of lesion reactivation would be good candidates for this approach. In a previous study, focal atrophy developing at the type 3 MNV lesion was associated with a low risk of lesion reactivation^27^. Based on this observation, we recommend that patients exhibiting focal RPE atrophy in the early treatment period can be candidates for starting with an as-needed regimen followed by switching to a TAE regimen.

The visual outcome in our patients using the as-needed regimen was relatively unfavorable with a mean visual deterioration of 1.5 lines, whereas a significant visual improvement was noted in a previous study by Shin et al^10^. despite a similar injection frequency. We believe the main reason for this difference is that we did not implement the standard as-needed regimen with monthly follow-up.^10^ Holz et al. demonstrated that less frequent hospital visits and injections are associated with a poor visual outcome in a real-world setting^28^. Thus, some of our patients might have been undertreated and this might have influenced the poor outcomes, especially in the as-needed group. Since not implementing the standard as-needed regimen with monthly follow-up is a major drawback of the present study, it should be considered when interpreting the study results. In the present study, patients who did not experience lesion reactivation were not included in the as-needed group and were analyzed separately. We postulate that this difference in the treatment protocol may have also influenced the difference in treatment outcomes between our patients and those in the study by Shin et al.^10^.

It is well-known that type 3 MNV involves a high risk of RPE atrophy development. The incidence is reported to be between 24 and 36.6% in long-term follow-up studies^7,8,29^. RPE atrophy is one of the primary reasons for visual deterioration in neovascular AMD^30^, and the development of fovea-involving RPE atrophy in type 3 MNV can have a negative impact on visual prognosis even when the macula remains dry. It is not clear whether an increased number of anti-VEGF injections facilitates the development or progression of RPE atrophy^31^. However, in a study by Matsumoto et al., the enlargement of RPE atrophy was positively associated with the number of anti-VEGF injections in type 3 MNV^11^, suggesting that the influence of anti-VEGF injections on RPE atrophy should not be completely neglected, at least in type 3 MNV.

Unlike the study by Matsumoto et al.^11^, the incidence of fovea-involving RPE atrophy was similar between the TAE and as-needed groups, despite the higher injection frequency in the TAE group, in the present study. We postulate that retinal damage caused by recurrent lesion reactivation in the as-needed group may have influenced the development of RPE atrophy and contributed to a similar incidence of RPE atrophy between the two groups. Recently, Sadda et al. underscored the importance of adequately treating neovascular AMD and avoiding undertreatment in a real-world setting^31^. We believe that the present study, which shows superior outcomes in the TAE group compared with the as-needed group, may support the suggestion of Sadda et al.^31^.

Nevertheless, there is one additional consideration. The proportion of our patients who underwent ranibizumab monotherapy throughout the entire follow-up period was relatively higher in the TAE group than in the as-needed group. Since the incidence of RPE atrophy can be higher for those treated using aflibercept rather than ranibizumab^32^, the usage of different anti-VEGF agents between the two groups may have had some influence on the RPE atrophy outcomes.

One interesting finding in the present study is that the visual outcome was relatively more favorable in the TAE group than in the no-reactivation group. We postulate that the primary reason for this finding is the markedly higher incidence of fovea-involving RPE atrophy in the no-reactivation group. In type 3 MNV, the development of RPE atrophy is reported to be associated with a low rate of lesion reactivation^27,33^. Thus, it is not surprising that the incidence of RPE atrophy is high in patients who did not experience lesion reactivation. Recently, Chong Teo et al. demonstrated that the visual outcome is better in moderate levels of disease activity than in higher or lower levels of disease activity^34^. The primary reason is suggested to be the development of subretinal fibrosis or macular atrophy in patients with lower levels of disease activity^34^. We believe that our result, a relatively favorable visual outcome in the TAE group compared with the no-reactivation group, aligns with the study results of Chong Teo et al.^34^.

Since the development or progression of RPE atrophy is associated with decreased lesion activity in type 3 MNV^27,33^, continuous TAE injections may lead to overtreatment in some patients with RPE atrophy. Several investigators have recently suggested to discontinue injections in patients with stable inactive disease who have received treatments at 16-week intervals for a few consecutive visits^35^. Kim et al. also propose that the development and progression of focal RPE atrophy can be considered a potential morphologic biomarker for discontinuing injections in type 3 MNV^27^. Determining the discontinuation of injections in type 3 MNV is difficult because both the possibility of unnecessary injections and the relentless nature of the disease itself should be equally considered. We hope further studies may provide more evidence to establish type 3 MNV-specific criteria for discontinuing injections.

In the present study, 132 patients were included. When a sample size calculation was performed with 95% confidence intervals and 80% test power, the necessary sample size for a Mann–Whitney *U* test for comparison between the TAE and as-needed groups was calculated as 216. The number of included patients was much less than the calculated value. For this reason, the small sample size is a limitation of the present study. However, due to the relative paucity of patients with type 3 MNV, including this number of patients was not possible in our single-center study. Further multi-center studies that enroll a sufficient number of patients are required.

In the TAE group, VAs measured when the treatment was changed from the as-needed regimen to the TAE regimen were slightly better than those measured at the corresponding time point in the as-needed group. However, in the TAE group, retinal fluid was not observed when the switching was performed, while in the as-needed group, patients with and without fluid were mixed at the time of VA measurement. We postulate that this difference had an influence on the slightly inferior VAs in the as-needed group.

The primary strength of the present study is that, to our knowledge, we provide the largest series comparing treatment outcomes between different treatment regimens in type 3 MNV. However, our study had obvious limitations. First, it was a retrospective study and the sample size was small. An additional limitation of the present study is that strict monthly follow-up was not performed when implementing the as-needed regimen. Therefore, some of our patients might have been undertreated. Second, in the TAE group, all patients were initially administered with as-needed retreatment and then switched to the TAE regimen. Thus, our study could not evaluate outcomes in patients who underwent the TAE regimen immediately after diagnosis. Third, different anti-VEGF agents were used for treatment, particularly in the as-needed group. To date, there is no firm evidence suggesting significant differences in the treatment efficacy of different agents for type 3 MNV. In addition, there was no difference in visual outcomes between the ranibizumab and aflibercept groups. However, the influence of different anti-VEGF agents on our results could not be ignored. Fourth, only patients who completed 24 months of follow-up were included in the study. Therefore, selection bias may have affected the study results. Fifth, since autofluorescence imaging was not routinely performed, the presence of RPE atrophy could not be accurately identified in some patients. Finally, all the patients were Korean.

In summary, the present study reveals that long-term visual outcomes of patients with type 3 MNV were markedly better in patients who were initially treated with the as-needed regimen but switched to the TAE regimen during treatment than those treated with the as-needed regimen throughout the follow-up period. In addition, the incidence of fibrotic scarring, RPE tears, and subretinal hemorrhage was relatively lower in the TAE group. Based on these observations, we suggest that retreatment based on the as-needed regimen during the early treatment period before switching to the TAE regimen would be a useful approach for patients with type 3 MNV requiring efficient treatment. However, since retinal damage due to recurrent reactivation can accumulate during the as-needed phase, further studies are required to better elucidate the proper timing of the switch of regimen to achieve the best treatment outcome.

## Data Availability

The datasets generated and/or analyzed during the current study are available with the corresponding author upon reasonable request.
